# Witnesses of causal nonseparability: an introduction and a few case studies

**DOI:** 10.1038/srep26018

**Published:** 2016-05-18

**Authors:** Cyril Branciard

**Affiliations:** 1 Institut Néel, CNRS and Université Grenoble Alpes, 38042 Grenoble Cedex 9, France

## Abstract

It was recently realised that quantum theory allows for so-called *causally nonseparable processes*, which are incompatible with any definite causal order. This was first suggested on a rather abstract level by the formalism of *process matrices*, an extension of the quantum formalism which only assumes that quantum theory holds locally in some observers’ laboratories, but does not impose a global causal structure; it was then shown, on a more practical level, that the *quantum switch*—a new, already implementable resource for quantum computation that goes beyond causally ordered circuits—provided precisely a physical example of a causally nonseparable process. To demonstrate that a given process is causally nonseparable, the concept of *witnesses of causal nonseparability* was introduced. Here we present a shorter introduction to this concept, and concentrate on some explicit examples—by considering in particular different noise models for the quantum switch—to show how to construct and use such witnesses in practice.

In our common understanding of the world, we typically perceive events as happening one after another, in a given order. Relations between events are understood in terms of causes and effects, where a cause can only precede an effect. Events can thus be embedded in a *causal structure*, which defines the *causal order* between them.

This viewpoint is ingrained for instance in the circuit model for computation or information processing, where operations are performed by gates that are applied in a definite order. While the assumption that events follow a definite causal order seems natural in the classical world, one may nevertheless wonder whether it must really always be so. One may in particular become suspicious when entering the quantum world, where the properties of physical systems are not always well-defined.

A general framework, that of *process matrices*, was recently introduced to investigate physical processes without pre-assuming a definite global causal structure; the framework only assumes that quantum theory correctly describes what happens locally, in some observers’ laboratories^1^. It was shown that this allows for processes that are incompatible with any definite causal order—so-called *causally nonseparable processes*. The framework was first introduced on a rather abstract level, with no clear physical interpretation given to the first examples of causally nonseparable processes. However, a concrete physical example of a causally nonseparable process was later exhibited^2^,^3^: namely, the recently proposed *quantum switch*, a new resource for quantum computation where the order of operations is controlled by a qubit in a superposition of two different states—which indeed does not fit in the standard framework of causally ordered quantum circuits^4^.

To ensure that this notion of causal nonseparability has any practical meaning, one needs of course to be able to verify that a given process is causally nonseparable. This was first done in ref. 1 through the violation of a *causal inequality*—an inequality bounding the correlations compatible with a definite causal order, and whose violation can only be obtained from a causally nonseparable process. This is however a very strong argument for causal nonseparability. In fact, not all causally nonseparable processes violate a causal inequality; the quantum switch indeed provides such an example^2^,^3^.

More recently we introduced, in analogy with entanglement witnesses, the concept of *witnesses of causal nonseparability* (or *causal witnesses*, as we initially called them)^2^. Here a witness corresponds to an operator that can (in principle) be ‘measured’ on a given process by combining the statistics of various operations, and whose expectation value, if negative, certifies the causal nonseparability of the process. We showed in particular that a witness can be efficiently constructed for any causally nonseparable process.

The objective of this paper is to present a somewhat shorter introduction to this new concept of witnesses of causal nonseparability. We will avoid here some of the technicalities in the proofs, and refer directly to ref. 2 for that. We will then present several different explicit examples of causally nonseparable processes and of witnesses—in particular for the quantum switch, investigating its robustness to different kinds of noise—so as to illustrate how to construct and use them in practice.

## The process matrix formalism

### In the general bipartite case

Consider an experiment with two parties, Alice and Bob, sitting in closed laboratories and exchanging physical systems. In a single run of the experiment, each party opens their lab only once to let some incoming system enter, and once to send some outgoing system out. They can perform some operation on these systems, which may output some result *a* for Alice and *b* for Bob.

While we do not pre-suppose a definite causal order between the events happening in Alice and Bob’s labs, we assume that what happens *locally* inside the labs is correctly described by quantum theory. That means, we can attach some Hilbert spaces 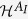 and 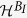 to their incoming systems and some Hilbert spaces 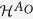 and 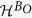 to their outgoing systems, and their choices of operations correspond to so-called quantum instruments^5^—i.e., sets of completely positive (CP) maps which sum up to CP and trace-preserving maps^6^. These can conveniently be represented, using the Choi-Jamiołkowski (CJ) isomorphism, by positive semidefinite matrices 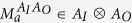 and 
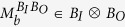
, where *A*_*I*_ and *A*_*O*_ (resp. *B*_*I*_ and *B*_*O*_) denote the spaces of Hermitian linear operators over Alice’s (Bob’s) incoming and outgoing Hilbert spaces, and where the subscripts refer to the outcomes *a*, *b* they correspond to. To define valid instruments, these matrices must satisfy


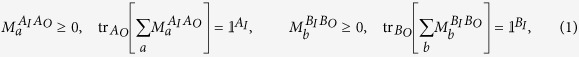


where 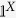
denotes the identity operator in the space *X* (in general, superscripts on operators will refer to the space they are acting on) and tr_*X*_ is the partial trace over *X*. In this paper we will only consider finite-dimensional Hilbert spaces; the dimension of a Hilbert space 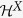 will be denoted *d*_*X*_.

#### Process matrices

**Figure 1:**
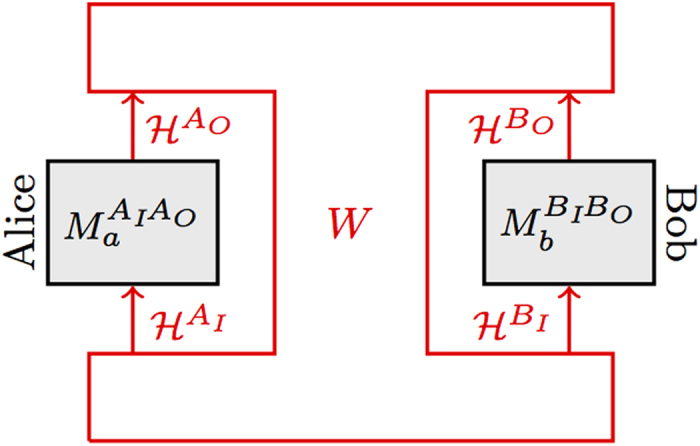
Two parties, Alice and Bob, perform some quantum operations 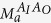 and 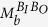
—some CP maps with outcomes *a*, *b*—which act on some incoming systems in the Hilbert spaces 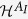, 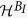 and generate some outgoing systems in the Hilbert spaces 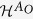, 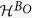. The *process matrix W* represents the physical resource that connects their labs, generalising the notions of quantum states and of quantum channels.

The correlations established by Alice and Bob in such a scenario can be described by the probabilities 
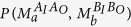
 that Alice and Bob obtain the outcomes *a*, *b* attached to the CP maps 
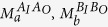
. As shown in^1^, these correlations can be written in the form


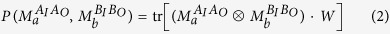


(with tr now denoting the full trace), for some Hermitian matrix 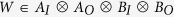. This so-called *process matrix* is the central object of the formalism; it describes the physical resource (the *process*) that connects Alice and Bob’s labs, and generalises both the notion of a quantum state—in which case Eq. (2) reduces to the standard Born rule—and of a quantum channel; see Fig. 1.

Not all matrices 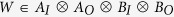 define valid processes. As one can show^1^,^2^, the constraint that all probabilities obtained through (2) must be nonnegative and normalised (including in situations where Alice and Bob may share and interact with ancillary entangled systems) leads to the following conditions that valid process matrices must satisfy:


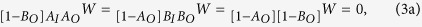



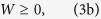



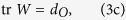


with 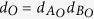 and where we used (and will use throughout the paper) the following notation, introduced in^2^:


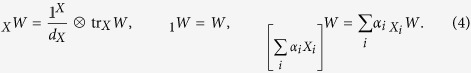


Equation (3a) defines a linear subspace 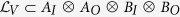, which valid process matrices belong to Eq. (3b) tells us that process matrices are in the set 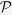 of positive semidefinite matrices. We shall often ignore, for convenience, the normalisation condition (3c), and define the set of nonnormalised process matrices as 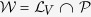; as can easily be checked, this set is a closed convex cone.

#### Causally separable vs causally nonseparable processes

Processes that do not allow Bob to signal to Alice are compatible with a causal order where Alice acts before Bob, which we write 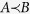. We shall generically denote by 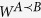 the corresponding process matrices; these simply represent standard, causally ordered quantum circuits. One can show that these are the matrices in 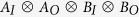, which satisfy^2^,^7^,^8^


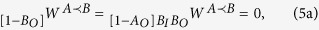



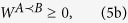



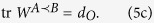


Note that Eq. (5a) implies Eq. (3a), which ensures that the 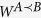 matrices thus characterised are valid process matrices. Equation (5a) thus defines a linear subspace 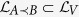. Together with Eq. (5b), we can define the closed convex cone of nonnormalised process matrices compatible with the causal order 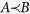, as 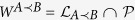.

Similarly, processes that do not allow Alice to signal to Bob are compatible with a causal order 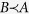, where Bob acts before Alice. The corresponding process matrices 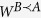 (which again simply represent standard, causally ordered quantum circuits) satisfy


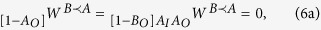



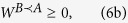



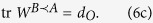


Equation (6a) defines a linear subspace 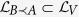. Together with Eq. (6b), we define the closed convex cone of nonnormalised process matrices compatible with the causal order 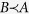, as 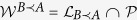.

One can still easily make sense of a convex mixture


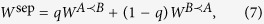


representing a process that is compatible with the causal order 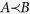 with some probability *q* ∈ [0, 1], and compatible with the causal order 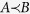 with some probability 1 − *q*. Process matrices that can be decomposed in this form (or directly, the process they represent) are said to be *causally separable*. Ignoring again the normalisation constraint, the set of nonnormalised causally separable process matrices also forms a closed convex cone, obtained as the Minkowski sum


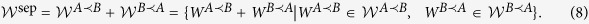


As first proven in^1^, there exist valid process matrices that *cannot* be decomposed as in (7), and which are therefore not in 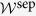. These are called *causally nonseparable*, and represent processes that are incompatible with any definite causal order—be it well-defined, or only determined with some probability.

### In a particular tripartite scenario

The scenario considered before can be generalised to more parties. While it is fairly easy to construct and characterise multipartite process matrices^1^,^2^,^9^ defining the notion of causal (non)separability is somewhat more subtle in such a setting^3^. In ref. 2 we restricted our study to a specific tripartite scenario, whose analysis matches that in the bipartite case quite closely (note indeed the similarities between the equations below and those in the previous subsection). We will again restrict ourselves to that case here, which is already quite relevant in practice, as we will see with the example of the quantum switch below.

#### Process matrices

In this particular scenario, the third party we introduce, Charlie, only has an incoming system in a Hilbert space 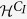 (as before, we will denote by 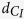 its dimension, and by *C*_*I*_ the space of Hermitian linear operators acting on 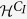), with no outgoing system—or equivalently: Charlie has a trivial outgoing system, in a trivial Hilbert space 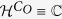 of dimension 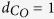. For a CP map 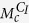 applied by Charlie, which reduces here to an element of a positive operator-valued measure (POVM)^2^,^6^ the generalised Born rule (2) simply becomes


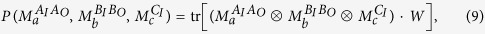


with now a process matrix 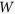 in 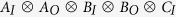.

Valid process matrices in this scenario satisfy^2^


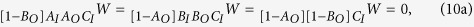



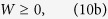



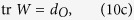


with again 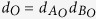. Equation (10a) defines, as before, a linear subspace 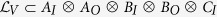. We can again characterise the closed convex cone of nonnormalised process matrices as 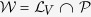.

#### Causally separable vs causally nonseparable processes

Since we assume that Charlie does not send any outgoing system out of his lab, one can argue^2^ that the only relevant causal orders are those where he is last; we are thus left to consider only the orders 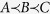 and 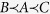.

The process matrices 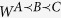 that are compatible with the causal order 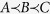 (and which thus, again, simply represent standard, causally ordered quantum circuits) are those, which satisfy^2^,^7^,^8^


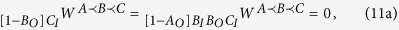



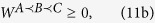



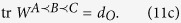


Equation (11a) defines here a linear subspace 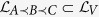. Together with Eq. (11b), we define the closed convex cone of nonnormalised process matrices compatible with the causal order 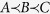, as 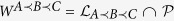.

Similarly, the process matrices 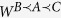 that are compatible with the causal order 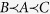 are those which satisfy


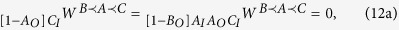



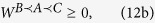



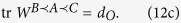


Equation (12a) defines a linear subspace 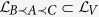. The closed convex cone of nonnormalised process matrices compatible with the causal order 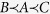 is defined here as 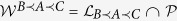.

In analogy with the previous case, any process matrix in the present scenario that can be decomposed as


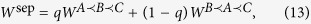


with *q* ∈ [0, 1], is called *causally separable*. (This definition of causal separability was proposed in^2^ for the particular tripartite case we consider here, which differs from that proposed in^3^ for general multipartite processes.) The set of nonnormalised causally separable process matrices also forms a closed convex cone, which can again be expressed here as the Minkowski sum


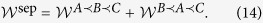


Process matrices that *cannot* be decomposed as in (13), and are thus not in 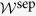, are called *causally nonseparable*. These are incompatible with any definite causal order (with Charlie last)—be it well-defined, or only determined with some probability.

## Witnesses of causal nonseparability

### Definition and characterisation

The concept of causal nonseparability represents a new type of resource compatible (at least locally) with quantum theory, which allows us to go beyond the standard framework of causally ordered quantum circuits^4^. An important question, to ensure this concept has some concrete physical ground, is: how to detect it and verify it in practice? One possible approach, used by Oreshkov *et al.* in^1^, is through the violation of a so-called *causal inequality*—namely, a bound on the correlations that are compatible with a definite causal order. Since all correlations generated by causally separable processes must satisfy such an inequality, a violation indeed ensures that the underlying process is causally nonseparable. Note that such a demonstration is *device-independent*, in the sense that one only looks at the observed correlations, without making assumptions on what operations the devices perform. Violating a causal inequality is however quite a strong requirement. In fact, just as not all entangled quantum states violate a Bell inequality^10^,^11^, not all causally nonseparable processes violate a causal inequality^2^,^3^ (an example being the quantum switch described below): one must then use less stringent criteria to detect causal nonseparability.

**Figure 2:**
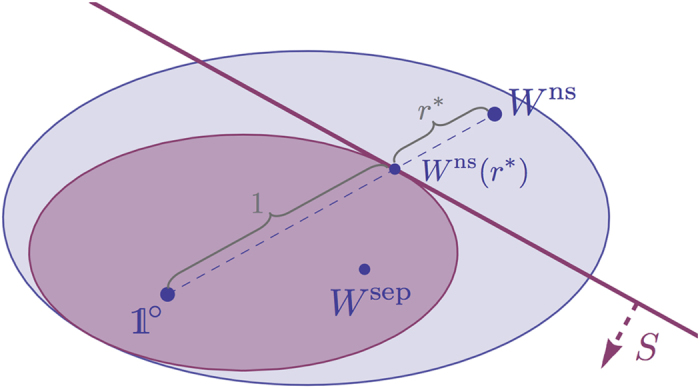
The set of causally separable process matrices, schematically represented by the inner ellipse, is closed and convex. From the separating hyperplane theorem, for any causally nonseparable process matrix 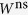 (in the larger ellipse containing all valid process matrices), there exists a hyperplane, represented by the solid line, that separates it from all causally separable process matrices 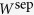. That is, there exists a Hermitian operator *S*—a *witness of causal nonseparability*—such that 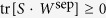 for all 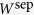, but 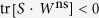. Solving the SDP problems presented below provides such a witness, which is optimal with respect to the resistance of 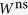 to white noise, represented by the process matrix 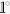: as depicted on the Figure, it detects the causal nonseparability of all process matrices 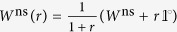 for *r* lower than the random robustness r* (directly obtained as a result of the SDP optimisation) above which 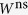
(*r*) becomes causally separable.

In ref. 2 we introduced for that, in analogy with entanglement witnesses^12^,^13^, the concept of *witnesses of causal nonseparability*—which we simply abbreviated (somewhat abusively) to *causal witnesses*. In this context, a witness is defined as any Hermitian operator *S* such that


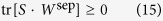


for all causally separable process matrices 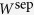. Since the set of causally separable process matrices is convex, then according to the separating hyperplane theorem^14^, for any causally nonseparable 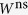 there must always exist a witness such that 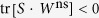, which can thus be used to certify the causal nonseparability of 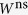; see Fig. 2. Note that the measurement of a witness is a *device-dependent* test of causal nonseparability, as the physical operations of the parties must faithfully realise *S* to be able to test Eq. (15).

According to the above definition, and considering the trace as the Hilbert–Schmidt inner product, the set *S* of witnesses of causal nonseparability is simply the *dual cone* (which we denote using an asterisk) of the cone of nonnormalised causally separable process matrices:


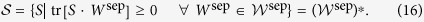


In the bipartite and particular tripartite cases considered here, this observation allows us to easily characterise the sets of witnesses 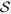, from the previous definitions of the corresponding cones 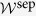; we do this explicitly in the Supplementary Information (SI), Part A, and report these characterisations in the Methods section below, for convenience.

Note that for any *S*^⊥^ in the orthogonal complement 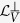 of the linear subspace 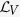, and for any valid process matrix *W* in 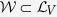, one has 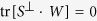. Hence, adding any term 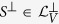 to a witness *S* simply gives another witness, giving the same value of tr[*S* · *W*] for any valid *W*. By choosing for instance *S*^⊥^ = *L*_*V*_(*S*) − *S*, where *L*_*V*_ is the projector onto the linear subspace 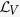, one thus obtains a witness in 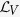. For practical reasons, we will often be led to restrict the search of witnesses within the subspace 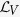; for that purpose we also define the (closed convex) cone of witnesses in 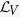 as 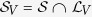.

### Determining causal (non) separability through semidefinite programming

To determine whether a given process is causally separable or not, one possible approach is to rephrase the question as an optimisation problem, and ask how much noise can be added before it becomes causally separable.

Let us consider for now the case of ‘white noise’, represented by the process matrix


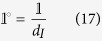


with 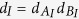 or 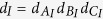 in the bipartite and tripartite cases, and which prepares the incoming systems of all parties in a maximally mixed state. For a given process matrix *W* under consideration, we shall consider the noisy process


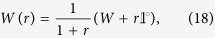


and investigate its causal nonseparability. Remembering that the normalisation of *W*(*r*) is irrelevant to check whether it is in the convex cone 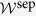 of causally separable processes, this leads us to define the following optimisation problem:


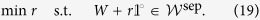


From the previous characterisation of the convex cone 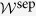, one can see that this defines a semidefinite programming (SDP) problem^15^, which can be solved efficiently. For ease of reference, we provide in the Methods section a more explicit description of this problem in terms of positive semidefinite constraints; see Eqs (52) and (54) for the bipartite and tripartite cases, respectively. As can be seen, solving this problem provides an explicit decomposition of *W*(r*), where r* is the optimal solution of (19), as a convex combination of processes 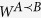 and 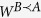. In analogy with the robustness of entanglement^16^, the quantity max[r*, 0] quantifies the robustness of the process *W* with respect to white noise—or *random robustness*^2^. In particular, a value r* > 0 implies that *W* is causally nonseparable.

The ‘primal’ SDP problem (19) is intimately linked to its ‘dual’ problem, which is here^2^


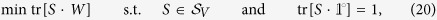


and whose optimal solution S* provides precisely, in the case where tr[S* · *W*] < 0, a witness of the causal nonseparability of *W*. Furthermore, the Duality Theorem for SDP problems^15^ implies that the solutions of the primal and dual problems satisfy


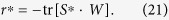


It follows in particular that tr[S* · *W*(*r*)] < 0 for all *r* < r*, i.e. for all *r* such that *W*(*r*) is causally nonseparable: this makes the witness S* optimal to detect the causal nonseparability of *W* when subjected to white noise, see Fig. 2.

As for the primal problem, we provide in the Methods section a more explicit description of the dual problem (20) that is better suited for practical use; see Eqs (53) and (54). It is worth noting that, as discussed previously, adding any term 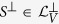 to *S* will not change the value of tr[*S* · *W*], nor of tr[*S* · 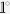]. Hence, the problem (20) is formally equivalent to one, where the constraint 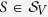 would be replaced by 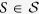; nevertheless, in practice, optimising over the whole (non-pointed) cone 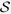 may make the numerical solvers unstable^2^.

Note that depending on the practical physical implementation of a process *W*, different noise models may also be relevant. One could consider for instance a mixture with another fixed process *W*°, and thus replace 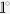 in the primal SDP problem (19) by *W*°. The normalisation constraint in the dual problem (20) would then be replaced by tr[*S* · *W*°] = 1 and one can show, following similar proofs to those of ref. 2, that as long as *W*° is in the relative interior of 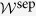 (i.e., the interior of 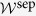 within 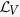), the SDP problems would still be solved efficiently, with their optimal solutions still satisfying (21).

Another case of interest is that of robustness to *worst case noise*, as also considered in ref. 2. One can define in this case the notion of *generalised robustness* (again in analogy with entanglement^17^), which can also be obtained through SDP. Interestingly, the generalised robustness can be used to define a proper *measure* of causal nonseparability as it is (contrary to the random robustness) monotonous under local operations^2^.

### Imposing further constraints on the witnesses

In order to ‘measure’ a witness *S*—i.e., to estimate the value tr[*S* · *W*] (and check its sign)—one can in principle simply decompose it as a linear combination of products of CP (trace non-increasing) maps, implement these maps (provided this can be done even if the causal order between the parties is not well-defined), estimate their probabilities, and combine the statistics in an appropriate way (as illustrated for instance in the next section)^2^.

In some cases, one may however not be able to implement all required CP maps, but may be restricted to CP maps from a certain class only—e.g., one may only be able to realise unitary operations. In that case, not all witnesses can be measured, and it then makes sense to restrict the search of witnesses to those that are implementable in practice. To do this, one can directly modify the dual problem (20) and replace the search space 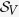 by the set 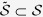 of allowed witnesses (while no longer necessarily restricting the search to witnesses within 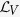).

Of course, with such an additional restriction the witnesses we shall obtain may not be optimal, and we will in general not be able to witness all causally nonseparable processes. Nevertheless, this possibility to add some constraints on the possible witnesses may be useful in practice, as we will illustrate below with the quantum switch.

## Case studies

Let us now consider a few concrete examples to illustrate how one can construct witnesses and characterise causal nonseparability in practice. We start with a family of bipartite processes investigated already in ref. 18, and then move on to the example of the quantum switch, for which we will consider different noise models and show how to add specific constraints on the witnesses we shall construct.

### A family of bipartite process matrices

In ref. 18, the following family of process matrices was introduced:


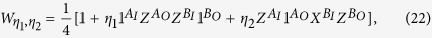


where *Z* and *X* are the Pauli matrices, the superscripts indicate to which system each operator is applied, and tensor products are implicit. 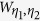 generalises in particular the process matrix originally considered in ref. 1, obtained for 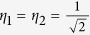. One can easily check that 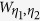 satisfies Eqs (3a) and (3c), and that it is positive semidefinite—hence, it is a valid process matrix—if and only if 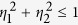.

We solved, for different values of *η*_1_, *η*_2_, the dual SDP problem (20)—or rather, its more explicit formulation given in (53)—using the Matlab software CVX^19^, and obtained (up to numerical precision) the witnesses


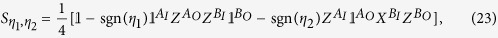


where sgn is the sign function (for 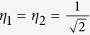 we recover the witness obtained in ref. 2).

To verify that 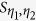 is indeed a valid witness, one can check that 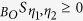 and 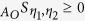: see the characterisation of witnesses in the bipartite case given in the Methods section, Eqs (43)–(44), 209. Applying 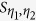 to 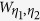, one gets


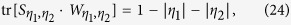


which shows that 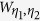 is causally nonseparable (the trace above is negative) for 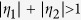, and its random robustness in that case is 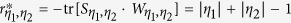.

For 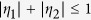 on the other hand, we find that 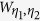 is causally separable. Solving the primal SDP problem (19)—or rather, its more explicit formulation (52)—provides an explicit decomposition as a convex sum of processes compatible with a definite causal order, in the form


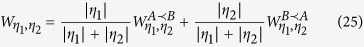


with


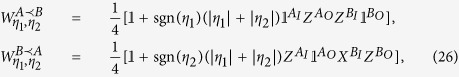


where one can indeed check that 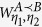 and 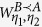 satisfy Eqs (5a)–(5c, 27, 28) and (6a)–(6c, 36, 37), as required (they are positive semidefinite precisely for 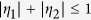).

**Figure 3:**
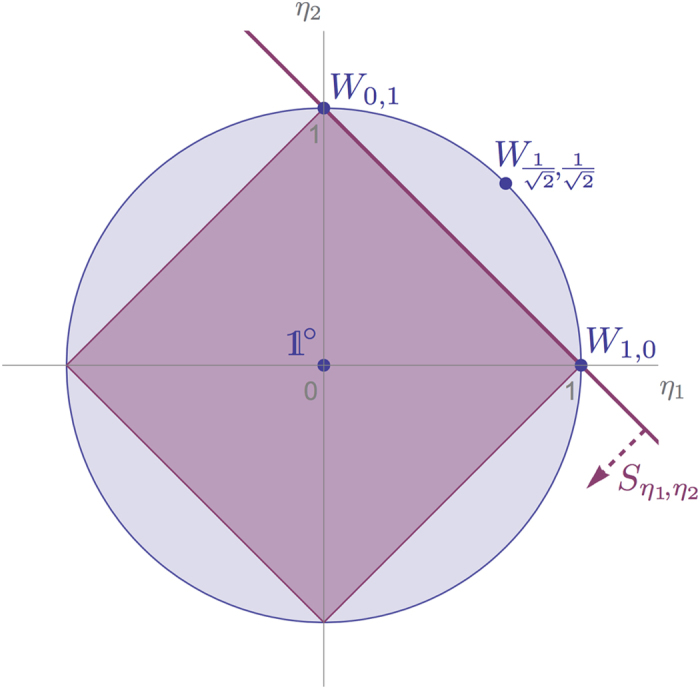
Representation of the set of process matrices 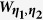 defined in Eq. (22). The shaded circle (characterised by 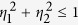) delimits the valid process matrices 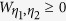. Causally separable processes 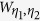 are restricted to the inner square (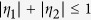). Causally nonseparable processes (such that 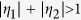) can be witnessed by 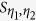 (23), represented (for the case *η*_1_, *η*_2_ ≥ 0) by the solid line. The figure here is similar to Fig. 2 of ref. 18.

Figure 3 represents the set of process matrices 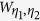. We recover here the results found in ref. 18; however, the use of witnesses allows us to give a much more direct proof of causal (non)separability for the 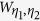 matrices.

In order to measure the witness 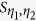 in practice, one can for instance decompose its two nontrivial components in terms of CP (trace non-increasing) maps as follows:


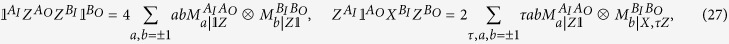


with


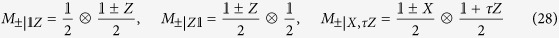


(where the second part of the subscripts denotes a particular choice of instrument: a choice of ‘setting’), and then calculate, using the generalised Born rule (2),


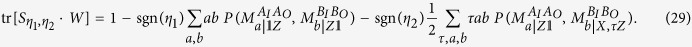


(Note that the decomposition of a witness in terms of CP maps is not unique; another possible decomposition of 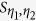, for the case *η*_1_, *η*_2_ > 0, was given in ref. 2).

### The quantum switch

The quantum switch is a circuit, which was originally proposed (independently from the framework of process matrices) to extend the framework of causally ordered quantum circuits and allow the order in which gates are performed to be coherently controlled by a quantum system^4^. As proven recently^2^,^3^, when analysed in the process matrix formalism, the quantum switch provides precisely an example of a (tripartite) causally nonseparable process. It is in fact the first practical example that we know how to realise physically (and which has been demonstrated experimentally^20^), as, to the best of our knowledge, no practical realisation is known so far for any of the causally nonseparable process matrices exhibited, e.g., in refs 1,9,18,21,22.

In its simplest version, the quantum switch involves two qubits—a control qubit and a target qubit. The target qubit, initially prepared in some state |*ψ*〉, is sent to two parties, Alice and Bob, who act on it in an order that is determined by the state of the control qubit: if the control qubit is in the state |0〉, then Alice acts first and Bob acts second, while if it is in the state |1〉, then Bob acts first and Alice second. The interesting situation is when the control qubit is in a superposition 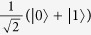, in which case Alice and Bob can be said to act ‘in a superposition of orders’. After Alice and Bob’s operations, the control qubit is sent to a third party, Charlie, who can measure it.

As shown in ref. 2 (see also^3^), the quantum switch can be represented in terms of the ‘pure process’


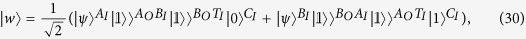


where |
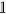
〉〉 = |00〉 + |11〉 is the CJ representation of an identity qubit channel, and which involves the incoming and outgoing systems *A*_*I*_, *A*_*O*_, *B*_*I*_ and *B*_*O*_ of Alice and Bob, the incoming system *C*_*I*_ of Charlie, and a system *T*_*I*_ to which the target qubit is given. After tracing out the latter, we obtain the process matrix of the quantum switch as


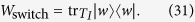


(Alternatively, the target qubit could also be sent for instance to Charlie, who could measure it together with the control qubit; for simplicity we do not consider this possibility here.) Note that 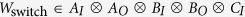 (with 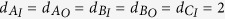) and that Charlie has no outgoing system, so that we are indeed in the particular tripartite case considered previously. It also appears clearly from Eq. (30) why we needed to introduce a third party in the description of the quantum switch, as tracing out *C*_*I*_ would otherwise result in a classical mixture of two causally ordered processes (i.e., in a causally separable process).

#### Robustness to white noise

To investigate the causal nonseparability of the quantum switch and construct a witness, one can follow the approach described previously. We solved the SDP problems (19)–(20)—or rather, their more explicit formulation (54)–(55)—numerically with CVX^19^, and found that the random robustness of the quantum switch is


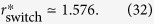


Alternatively, in terms of the ‘visibility’ *v*, this means that the noisy quantum switch


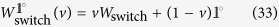


is causally nonseparable for all 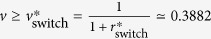. The explicit witness *S*_switch_ obtained numerically from the dual SDP problem (20) is given in SI, Part B.1.

#### Depolarising the control qubit

In a practical implementation of the quantum switch, other noise models than fully white noise can also be relevant.

Consider for instance a situation where, for practical reasons, the target qubit is well preserved throughout the setup, but the control qubit is affected by white noise: with some probability *v* (which can be understood as a ‘visibility’), the state of the control qubit is untouched, and with some probability 1 − *v* it is depolarised to the fully fixed state 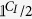. The resulting noisy process then writes


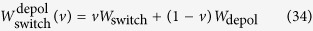


with


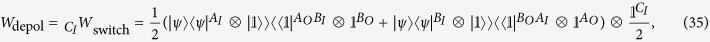


which corresponds to a random mixture of a process where the target qubit goes first to Alice then to Bob, and a process where it goes first to Bob and then to Alice.

One clearly sees that *W*_depol_ is causally separable. As it turns out, it lies precisely on the boundary of the set of causally separable processes; hence, some care needs to be taken if one wants to investigate the causal (non)separability of 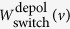 as discussed on page 6. A possible approach is for instance to mix the quantum switch with a process that is 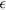-close to 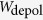 (and let 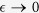), inside the relative interior of 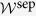; or to directly calculate the random robustness of 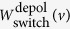 for various fixed values of *v*.

By doing so, we found numerically a positive random robustness for all chosen values *v* > 0. In fact, one can prove analytically that 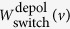 is causally nonseparable whenever *v* > 0, by constructing a family of witnesses *S*(*v*) such that 
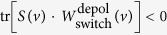
 for all *v* > 0; see SI, Part B.2. That is, the causal nonseparability of the quantum switch is infinitely robust to white noise affecting the control qubit only.

**Figure 4:**
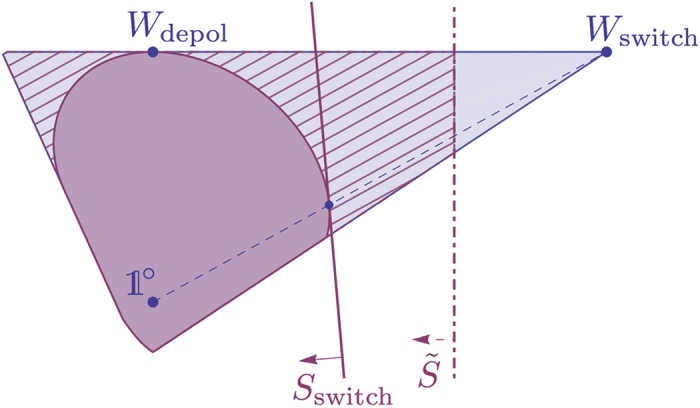
Two-dimensional slice of the space of process matrices containing *W*_switch_, *W*_depol_ and 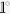. The shaded region contains all valid (positive semidefinite) process matrices, with the inner darker region containing the causally separable processes. The causal nonseparability of *W*_switch_ can be witnessed using *S*_switch_, given explicitly in SI, Part B.1, which is optimal to test its robustness to white noise. All processes 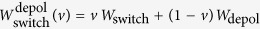 with 0 < *v* ≤ 1 are causally nonseparable, as can be shown using a family of witnesses given in SI, Part B.2. The witness 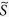 can be measured with Alice and Bob restricting their operations to unitaries; only the causally nonseparable processes outside of the hatched region can be witnessed with this restriction.

Figure 4 shows, for illustration, the two-dimensional slice of the space of process matrices that contains *W*_switch_, *W*_depol_ and 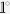. By scanning this whole slice, one can characterise using our SDP technique the limits of the set of causally separable processes. One can clearly see for instance that the whole line segment containing the processes 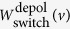 with *v* > 0 is outside of it, and approaches it tangentially.

#### Dephasing the control qubit

Rather than fully depolarising the control qubit, it may be relevant to investigate the case where it is only dephased, i.e. it undergoes (with some probability 1 − *v*, as before) the map


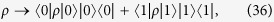


so that its coherence is lost.

We are thus led to consider here the noisy process


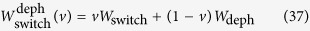


with


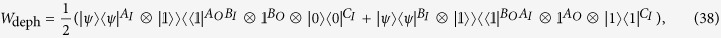


which corresponds now to a situation where a classical control bit, in the state 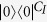 or 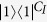 with equal probability, determines the order between Alice and Bob—a process that we could call a *classical switch*.

**Figure 5:**
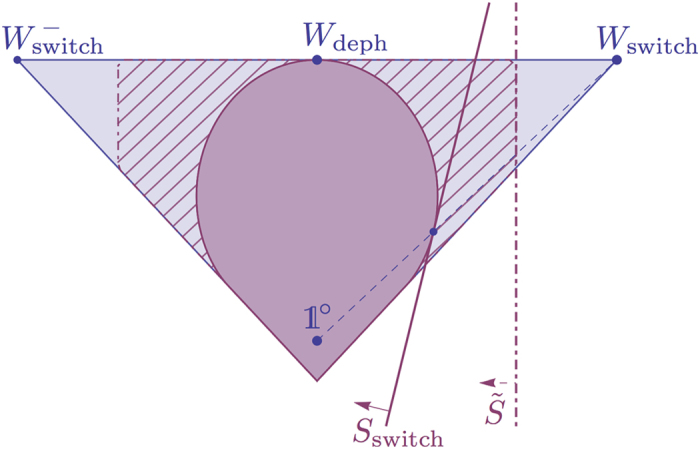
Analogous figure to Fig. 4, for the two-dimensional slice of the space of process matrices containing now *W*_switch_, *W*_deph_ and 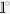. The process 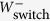, symmetric to *W*_switch_, is the process obtained when implementing the quantum switch with a control qubit initially in the state 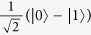 rather than 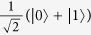 (whose description as a process matrix is then obtained by replacing the ‘+’ sign by a ‘−’ sign in Eq. (30)).

Clearly, *W*_deph_ is causally separable. Like *W*_depol_, it also lies on the boundary of the set of causally separable processes. One can again check numerically and prove analytically (see SI, Part B.2) that 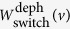 is causally nonseparable for all *v* > 0: that is, the quantum switch is also infinitely robust to dephasing noise affecting the control qubit only. As with Figs 4 and 5 now shows, for illustration, the two-dimensional slice of the space of process matrices that contains *W*_switch_, *W*_deph_ and 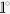.

#### Restricting Alice and Bob’s operations to unitaries

To finish with, let us consider an implementation of the quantum switch where Alice and Bob are restricted to perform unitary operations. This restriction is motivated by practical reasons: in the recent photonic implementation of the quantum switch reported in ref. 20 for example, Alice and Bob only used passive optical elements, namely half and quarter wave plates, realising (up to experimental imperfections) unitaries on the target qubit, encoded in the photon polarisation. In particular, Alice and Bob do not perform any actual measurement, and do not need to record measurement outcomes (only Charlie makes a measurement with different possible outcomes).

As we show in SI, Part B.3, the CJ representation 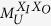 of a unitary operation 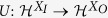 satisfies


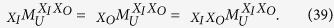


Now, if Alice and Bob are restricted to perform unitary operations, the witnesses that can be measured must be of the form


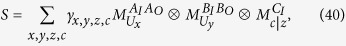


for some unitaries *U*_*x*_, *U*_*y*_, for some CP maps (or simply: POVM elements) 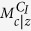, and some real coefficients *γ*_*x*,*y*,*z*,*c*_. Because of (39), *S* will then necessarily satisfy


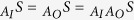


and


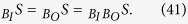


Hence, to construct such a witness, one can simply solve the dual SDP problem (20), replacing the constraint 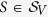 by 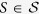 and Eq. (53). The resulting optimisation problem remains a SDP problem. Solving it with CVX, we obtained numerically an explicit witness 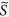, given in SI, Part B.3 and shown on Figs 4 and 5, that detects the causal nonseparability of the processes 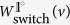 (33), 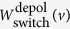 (34) and 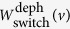 (37) down to *v* ≃ 0.6641 (the same value for all three).

Clearly, the price to pay by restricting Alice and Bob to unitaries only is that not all causally nonseparable processes can be witnessed; see the hatched regions in Figs 4 and 5. Nevertheless, the amount of noise tolerated by 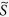 is already good enough to measure it and demonstrate causal nonseparability experimentally with current technologies, e.g. in a setup similar to that of ref. 20.

## Discussion

In this paper we have given an introduction to witnesses of causal nonseparability^2^, and illustrated this concept on a few explicit examples. Witnesses of causal nonseparability are somewhat analogous to entanglement witnesses; however, a remarkable difference is that contrary to the latter, the former can be constructed efficiently, for any causally nonseparable processes (in the bipartite and particular tripartite cases considered here), using semidefinite programming.

Among the explicit examples given above, of particular interest is the quantum switch. This is indeed the first concrete example of a causally nonseparable process that we know how to realise in practice, and for which we know how to witness the causal nonseparability. We constructed its optimal witness with respect to white noise, which detects its causal nonseparability down to a visibility of *v* ≃ 0.3882. We further constructed a witness that can be measured with Alice and Bob implementing unitaries only, and which is robust to visibilities down to *v* ≃ 0.6441—whether we consider white noise, or depolarising or dephasing noise that affects the control qubit only. This allows for a feasible experimental verification of the causal nonseparability of the quantum switch that would be more robust than with the witness previously proposed in^2^, which allows only for visibilities down to *v* ≃ 0.7381 (corresponding to a success probability 
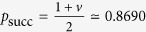
 for Chiribella’s task^23^, as reported in^2^). Note that in the latter, Charlie only performs measurements in the *X* basis (while our witness also involves the *Y* basis, see SI, Part B.3); as it turns out, that witness was actually optimal under this restriction, as can be shown by further adding the corresponding constraint in the dual problem (20). Recall that the witness obtained in^2^ was constructed from Chiribella’s task of distinguishing between a commuting and an anticommuting channel, where the quantum switch provides an advantage over any causally ordered circuit^23^. We note indeed that the tool of witnesses of causal nonseparability and the techniques developed to construct them may also be useful to inspire and analyse possible applications of causally nonseparable processes^23^,^24^,^25^, and to quantify their advantages over causally separable resources.

Let us finish by emphasising that in this paper, as in ref. 2, we only considered the bipartite case and a particular tripartite case, where the third party has no (or a trivial) outgoing system. Characterising and constructing witnesses in the general case remains so far an open problem. Clearly, the sets of nonnormalised process matrices and of witnesses remain closed convex cones, and one can still write the optimisation problems (19) and (20) as conic problems. However, whether the characterisation of the cones 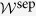 and 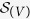 would allow us to write them as SDP problems that can be solved efficiently, and whether the duality relation (21) would still hold, is left for future research.

## Methods

### Characterisation of the cones *W*^sep^, *S* and *S*_*v*_

We recall in the Supplementary Information, Part A how to characterise the cones 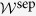 of (nonnormalised) causally separable process matrices, and the cones 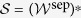 and 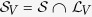 of witnesses of causal nonseparability, in the bipartite and particular tripartite cases considered in our paper (as was done previously in^2^). For ease of reference we report these characterisations here; this will be useful below to give more explicit forms for our SDP problems (19) and (20). It will be implicit here that all matrices under consideration are Hermitian, either in 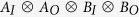 or in 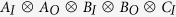; we will denote by 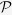 the cone of positive semidefinite matrices in either space.

#### Bipartite case

In the bipartite case, the cone of (nonnormalised) causally separable process matrices can be characterised as


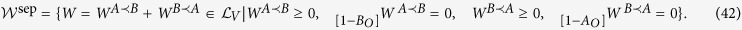


The cones 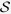 and 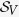 of witnesses of causal nonseparability can then be characterised as


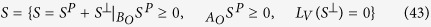


and


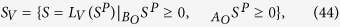


where *L*_*V*_ is the projector onto the subspace 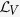, defined by


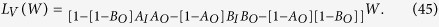


#### Tripartite case with 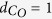


In the particular tripartite case where Charlie has a trivial outgoing system (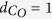), the cone of (nonnormalised) causally separable process matrices can similarly be written as


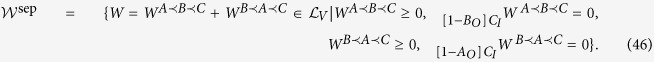


The cones 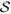 and 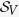 of witnesses of causal nonseparability can be characterised here as


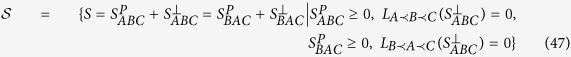


and


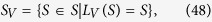


with the projectors 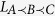, 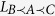 and *L*_*V*_ now defined by


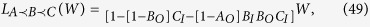



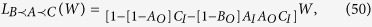



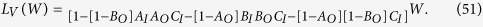


### Explicit formulation of our SDP problems

The previous characterisations of the cones 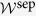 and 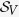 allow us to write (in our bipartite and tripartite cases) the primal and dual SDP problems (19) and (20) in more explicit forms, which can readily be implemented and solved on a computer.

#### Bipartite case

Using the characterisation of Eq. (42), and noting that for 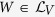, 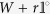 is also automatically in 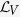, one can write explicitly the primal SDP problem (19) in the bipartite case as


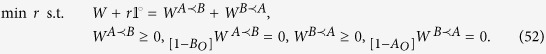


Using now Eq. (44), the dual SDP problem (20) writes, more explicitly,


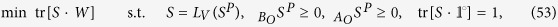


with *L*_*V*_ defined in Eq. (45).

#### Tripartite case with 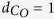


Using Eq. (46), the primal SDP problem (19) can similarly be written, in the tripartite case with 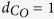, as


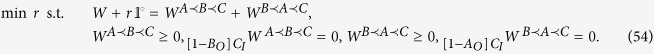


Using now Eqs (47)–(48), 218, the dual SDP problem (20) writes, more explicitly,


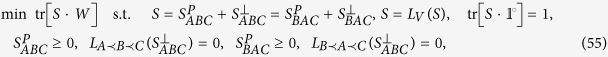


with 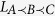, 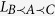 and 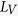 defined in Eqs (49)–(51), 222, 223.

## Additional Information

**How to cite this article**: Branciard, C. Witnesses of causal nonseparability: an introduction and a few case studies. *Sci. Rep.* **6**, 26018; doi: 10.1038/srep26018 (2016).

## Supplementary Material

Supplementary Information
