# Supplementary material for: Witnesses of causal nonseparability: an introduction and a few case studies
Source: Sci Rep. 2016 May 18;6:26018. doi: 10.1038/srep26018 (PMC4870702; doi:10.1038/srep26018)
Supplement: Supplementary Information [file srep26018-s1.pdf]

Supplementary Information for: Witnesses of causal nonseparability: an introduction and a few case studies

Cyril Branciard[1,*]

[1]Institut Néel, CNRS and Université Grenoble Alpes, 38042 Grenoble Cedex 9, France
[*]cyril.branciard@neel.cnrs.fr

In this Supplementary Information we show how to obtain the explicit characterisation of the cones $\mathcal{W}^{\text{sep}}$, $\mathcal{S}$ and $\mathcal{S}_V$ given in the Methods section (Part A), and we provide explicit witnesses for the quantum switch (Part B).

A Characterisation of the cones $\mathcal{W}^{\text{sep}}$, $\mathcal{S}$ and $\mathcal{S}_V$

Here we show how to explicitly characterise the cones $\mathcal{W}^{\text{sep}}$ of (nonnormalised) causally separable process matrices, and the cones $\mathcal{S} = (\mathcal{W}^{\text{sep}})^*$ and $\mathcal{S}_V = \mathcal{S} \cap \mathcal{L}_V$ of witnesses of causal nonseparability, in the bipartite and particular tripartite cases considered in the main text. The characterisations below were first obtained in Ref.[1] In what follows here, it is implicit that all matrices under consideration are either in $A_I \otimes A_O \otimes B_I \otimes B_O$ (in the bipartite case) or $A_I \otimes A_O \otimes B_I \otimes B_O \otimes C_I$ (in the tripartite case); in particular, they are all Hermitian.

A.1 $\mathcal{W}^{\text{sep}}$: Causally separable process matrices

A.1.1 Bipartite case

According to Eqs. (8), (5a)–(5b) and (6a)–(6b), bipartite causally separable process matrices can be written as

$$W = W^{A\prec B} + W^{B\prec A} \tag{S1}$$

with $W^{A\prec B}$ and $W^{B\prec A}$ two positive semidefinite matrices satisyfing

$$_{[1-B_O]}W^{A\prec B} = 0, \tag{S2a}$$
$$_{[1-A_O]B_I B_O}W^{A\prec B} = 0, \tag{S2b}$$
$$_{[1-A_O]}W^{B\prec A} = 0, \tag{S2c}$$
$$_{[1-B_O]A_I A_O}W^{B\prec A} = 0. \tag{S2d}$$

Note that if W is already assumed to be a valid process matrix in $\mathcal{L}_V$ (hence from Eq. (3a), it satisfies in particular $_{[1-B_O]A_I A_O}W = {}_{[1-A_O]B_I B_O}W = 0$), then assuming that $W^{A\prec B}$ satisfies (S2a) automatically implies that $W^{B\prec A} = W - W^{A\prec B}$ satisfies (S2d); similarly, assuming that $W^{B\prec A}$ satisfies (S2c) automatically implies that $W^{A\prec B}$ satisfies (S2b). Hence, to determine whether $W \in \mathcal{L}_V$ is causally separable, it is enough to check whether it can be decomposed as in (S1) with $W^{A\prec B} \geq 0$ and $W^{B\prec A} \geq 0$ satisfying (S2a) and (S2c), resp. Defining the linear subspaces

$$\mathcal{L}_{[1-B_O]} = \{W \,|\, _{[1-B_O]}W = 0\}, \qquad \mathcal{L}_{[1-A_O]} = \{W \,|\, _{[1-A_O]}W = 0\}, \tag{S3}$$

the cone of (nonnormalised) causally separable process matrices can then be characterised as[1]

$$\mathcal{W}^{\text{sep}} = \mathcal{W}^{A\prec B} + \mathcal{W}^{B\prec A} \tag{S4}$$
$$= \big[(\mathcal{P}\cap\mathcal{L}_{[1-B_O]}) + (\mathcal{P}\cap\mathcal{L}_{[1-A_O]})\big] \cap \mathcal{L}_V\,. \tag{S5}$$

This characterization is indeed equivalent to that of Eq. (42) given in the Methods section. (Note furthermore that in Ref.,[1] instead of using the Minkowski sum notation, we wrote equivalently $\mathcal{W}^{\text{sep}} = \text{conv}(\mathcal{W}^{A\prec B} \cup \mathcal{W}^{B\prec A})$, where conv denotes the convex hull.)

A.1.2 Tripartite case with $d_{C_O} = 1$

With similar arguments as in the bipartite case above, we find that in the particular tripartite case where Charlie has a trivial outgoing system ($d_{C_O} = 1$), the cone of (nonnormalised) causally separable process matrices can be characterised as

$$\mathcal{W}^{\text{sep}} = \mathcal{W}^{A\prec B\prec C} + \mathcal{W}^{B\prec A\prec C} \tag{S6}$$
$$= \big[(\mathcal{P}\cap\mathcal{L}_{[1-B_O]C_I}) + (\mathcal{P}\cap\mathcal{L}_{[1-A_O]C_I})\big] \cap \mathcal{L}_V \tag{S7}$$

with

$$\mathcal{L}_{[1-B_O]C_I} = \{W \,|\, {}_{[1-B_O]C_I}W = 0\}, \qquad \mathcal{L}_{[1-A_O]C_I} = \{W \,|\, {}_{[1-A_O]C_I}W = 0\}. \tag{S8}$$

This characterization is indeed equivalent to that of Eq. (46).

A.2 $\mathcal{S}$ and $\mathcal{S}_V$: Witnesses of causal nonseparability

As explained in the main text, the set of witnesses of causal nonseparability is simply the dual cone of $\mathcal{W}^{\rm sep}$. It can be characterised by using the previous descriptions of $\mathcal{W}^{\rm sep}$, and making use of the following duality relations for two nonempty closed convex cones $\mathcal{K}_1, \mathcal{K}_2$:[2]

$$(\mathcal{K}_1 + \mathcal{K}_2)^* = \mathcal{K}_1^* \cap \mathcal{K}_2^*, \qquad (\mathcal{K}_1 \cap \mathcal{K}_2)^* = \mathcal{K}_1^* + \mathcal{K}_2^*. \tag{S9}$$

A.2.1 *Bipartite case*

Using (S5) and (S9), noting that the dual cone of a linear subspace $\mathcal{L}$ is its orthogonal complement $\mathcal{L}^\perp$ and that the cone $\mathcal{P}$ of positive semidefinite matrices is self-dual, one can write, in the bipartite case,

$$\mathcal{S} = (\mathcal{W}^{\rm sep})^* = \big[(\mathcal{P} + \mathcal{L}_{[1-B_O]}^\perp) \cap (\mathcal{P} + \mathcal{L}_{[1-A_O]}^\perp)\big] + \mathcal{L}_V^\perp. \tag{S10}$$

Noting now that $\mathcal{L}_{[1-B_O]}^\perp = \{S \,|\, {}_{[1-B_O]}S = S\} = \{S \,|\, {}_{B_O}S = 0\}$ and that the map $S \to {}_{B_O}S$ is positive, one can easily show[1] that $\mathcal{P} + \mathcal{L}_{[1-B_O]}^\perp = \{S \,|\, {}_{B_O}S \geq 0\}$, and similarly that $\mathcal{P} + \mathcal{L}_{[1-A_O]}^\perp = \{S \,|\, {}_{A_O}S \geq 0\}$. Furthermore, one has $\mathcal{L}_V^\perp = \{S | L_V(S) = 0\}$, where L_V is the projector onto the linear subspace $\mathcal{L}_V (= \{S | L_V(S) = S\})$, which can be written as

$$\begin{aligned} L_V(W) &= {}_{[1-[1-B_O]A_IA_O]\,[1-[1-A_O]B_IB_O]\,[1-[1-A_O][1-B_O]]}W \\ &= {}_{[1-[1-B_O]A_IA_O-[1-A_O]B_IB_O-[1-A_O][1-B_O]]}W. \end{aligned} \tag{S11}$$

Combining this with (S10), we find that

$$\mathcal{S} = \Big\{ S = S^P + S^\perp \,\Big|\, {}_{B_O}S^P \geq 0, \; {}_{A_O}S^P \geq 0, \; L_V(S^\perp) = 0 \Big\}. \tag{S12}$$

Furthermore, $S = S^P + S^\perp$ thus characterised is in $\mathcal{L}_V$ if and only if $S = L_V(S_P + S^\perp) = L_V(S_P)$. Hence, we also simply have

$$\mathcal{S}_V = \Big\{ S = L_V(S^P) \,\Big|\, {}_{B_O}S^P \geq 0, \; {}_{A_O}S^P \geq 0 \Big\}. \tag{S13}$$

A.2.2 *Tripartite case with $d_{C_O} = 1$*

One could follow a similar reasoning as above to characterise $\mathcal{S}$ in the tripartite case with $d_{C_O} = 1$, starting from the characterisation of $\mathcal{W}^{\rm sep}$ given by Eq. (S7). However, because the map $S \to {}_{[1-(1-B_O)C_I]}S$ is not positive (contrary to $S \to {}_{[1-(1-B_O)]}S = {}_{B_O}S$), one cannot simplify the characterisation of $\mathcal{P} + \mathcal{L}_{[1-B_O]C_I}^\perp$—and ultimately of $\mathcal{S}$—as much as before.

It is thus somewhat simpler here to start directly from the characterisation of $\mathcal{W}^{\rm sep}$ given by Eq. (S6). With $\mathcal{W}^{A\prec B\prec C} = \mathcal{P} \cap \mathcal{L}_{A\prec B\prec C}$ and $\mathcal{W}^{B\prec A\prec C} = \mathcal{P} \cap \mathcal{L}_{B\prec A\prec C}$, we get, using again the relations (S9),

$$\begin{aligned} \mathcal{S} &= (\mathcal{W}^{\rm sep})^* = \big[(\mathcal{P} \cap \mathcal{L}_{A\prec B\prec C}) + (\mathcal{P} \cap \mathcal{L}_{B\prec A\prec C})\big]^* = (\mathcal{P} + \mathcal{L}_{A\prec B\prec C}^\perp) \cap (\mathcal{P} + \mathcal{L}_{B\prec A\prec C}^\perp) \\ &= \Big\{ S = S_{ABC}^P + S_{ABC}^\perp = S_{BAC}^P + S_{BAC}^\perp \,\Big|\, S_{ABC}^P \geq 0, \; L_{A\prec B\prec C}(S_{ABC}^\perp) = 0, \\ &\qquad\qquad S_{BAC}^P \geq 0, \; L_{B\prec A\prec C}(S_{ABC}^\perp) = 0 \Big\}, \end{aligned} \tag{S14}$$

where $L_{A\prec B\prec C}$ and $L_{B\prec A\prec C}$ are the projectors onto the linear subspaces $\mathcal{L}_{A\prec B\prec C}$ and $\mathcal{L}_{B\prec A\prec C}$, which are

$$L_{A\prec B\prec C}(W) = {}_{[1-[1-B_O]C_I-[1-A_O]B_IB_OC_I]}W, \qquad L_{B\prec A\prec C}(W) = {}_{[1-[1-A_O]C_I-[1-B_O]A_IA_OC_I]}W. \tag{S15}$$

Restricting the witnesses to the subspace $\mathcal{L}_V$, one can then write

$$\mathcal{S}_V = \{ S \in \mathcal{S} \,|\, L_V(S) = S \} \tag{S16}$$

by referring to the previous characterisation (S14) of $\mathcal{S}$, and with the projector L_V onto $\mathcal{L}_V$ now given by

$$L_V(W) = {}_{[1-[1-B_O]A_IA_OC_I-[1-A_O]B_IB_OC_I-[1-A_O][1-B_O]C_I]}W. \tag{S17}$$

B Witnesses for the quantum switch

In this second part we give explicit witnesses of the causal nonseparability of the quantum switch. Although the results reported in the main text do not depend on the initial state $|\psi\rangle$ of the target qubit, the specific form of the witnesses does; in the following we fix it to be $|\psi\rangle = |0\rangle$.

For ease of notations, we will provide the various witnesses in the general form

$$S = \frac{1}{4}\Big(\mathbb{1} + \sum_i s_i\, S_i\Big)\,, \tag{S18}$$

for some terms S_i and coefficients s_i to be specified below. To verify that S is a valid witness, we will provide the explicit decomposition of $S = S^P_{ABC} + S^\perp_{ABC}$ as in (S14) in the form

$$S^\perp_{ABC} = \frac{1}{4}\sum_j t_j\, T_j\,, \qquad S^P_{ABC} = S - S^\perp_{ABC}\,, \tag{S19}$$

for some terms T_i and coefficients t_i to be specified as well. This will allow the reader to check that $L_{A\prec B\prec C}(S^\perp_{ABC}) = 0$ and $S^P_{ABC} \geq 0$, as required by (S14).

Due to the symmetries of the quantum switch and its witnesses, the second decomposition $S = S^P_{BAC} + S^\perp_{BAC}$ in (S14) can then be obtained as

$$S^\perp_{BAC} = \mathcal{F}_{A\leftrightarrow B}(S^\perp_{ABC})\,, \qquad S^P_{BAC} = \mathcal{F}_{A\leftrightarrow B}(S^P_{ABC})\,, \tag{S20}$$

where $\mathcal{F}_{A\leftrightarrow B}$ is the map that exchanges the roles of Alice and Bob, defined as

$$\mathcal{F}_{A\leftrightarrow B}(\sigma_1^{A_I}\otimes\sigma_2^{A_O}\otimes\sigma_3^{B_I}\otimes\sigma_4^{B_O}\otimes\sigma_5^{C_I}) = \pm\,\sigma_3^{A_I}\otimes\sigma_4^{A_O}\otimes\sigma_1^{B_I}\otimes\sigma_2^{B_O}\otimes\sigma_5^{C_I} \tag{S21}$$

for $\sigma_i = \mathbb{1}, X, Y, Z$, and where the sign is $+$ if $\sigma_5 = \mathbb{1}$ or X, and $-$ if $\sigma_5 = Y$ or Z. (Note that all processes W and all witnesses S considered for the tripartite case in this paper have the symmetry $W = \mathcal{F}_{A\leftrightarrow B}(W)$, $S = \mathcal{F}_{A\leftrightarrow B}(S)$.)

B.1 Optimal witness with respect to white noise

By solving the dual SDP problem (55) for $W = W_{\text{switch}}$ with CVX, we obtained numerically the witness S_{switch} of the form (S18), with

$$\begin{aligned}
S_1 &= Z\mathbb{1}Z\mathbb{1}\mathbb{1}\,, \qquad S_2 = Z\mathbb{1}\mathbb{1}\mathbb{1}\mathbb{1} + \mathbb{1}\mathbb{1}Z\mathbb{1}\mathbb{1}\,, \qquad S_3 = \mathbb{1}ZZ\mathbb{1}\mathbb{1} + Z\mathbb{1}\mathbb{1}Z\mathbb{1}\,,\\
S_4 &= ZZZ\mathbb{1}\mathbb{1} + Z\mathbb{1}ZZ\mathbb{1}\,, \qquad S_5 = ZZ\mathbb{1}\mathbb{1}Z - \mathbb{1}\mathbb{1}ZZZ\,, \qquad S_6 = \mathbb{1}ZZ\mathbb{1}Z - Z\mathbb{1}\mathbb{1}ZZ\,,\\
S_7 &= Z\mathbb{1}\mathbb{1}\mathbb{1}Z - \mathbb{1}\mathbb{1}Z\mathbb{1}Z + ZZ\mathbb{1}ZZ - \mathbb{1}ZZZZ\,, \qquad S_8 = \mathbb{1}Z\mathbb{1}\mathbb{1}Z - \mathbb{1}\mathbb{1}\mathbb{1}ZZ + ZZZ\mathbb{1}Z - Z\mathbb{1}ZZZ\,,\\
S_9 &= \mathbb{1}X\mathbb{1}XX + \mathbb{1}Y\mathbb{1}YX + \mathbb{1}X\mathbb{1}YY - \mathbb{1}Y\mathbb{1}XY + \mathbb{1}XZXX + \mathbb{1}YZYX + \mathbb{1}XZYY - \mathbb{1}YZXY\\
&\quad + ZX\mathbb{1}XX + ZY\mathbb{1}YX + ZX\mathbb{1}YY - ZY\mathbb{1}XY + ZXZXX + ZYZYX + ZXZYY - ZYZXY\,,\\
S_{10} &= X\mathbb{1}X\mathbb{1}X - X\mathbb{1}XZX - XZX\mathbb{1}X + XZXZX + Y\mathbb{1}Y\mathbb{1}X - Y\mathbb{1}YZX - YZY\mathbb{1}X + YZYZX\\
&\quad + X\mathbb{1}Y\mathbb{1}Y - X\mathbb{1}YZY - XZY\mathbb{1}Y + XZYZY - Y\mathbb{1}X\mathbb{1}Y + Y\mathbb{1}XZY + YZX\mathbb{1}Y - YZXZY\,,\\
S_{11} &= \mathbb{1}XX\mathbb{1}\mathbb{1} - \mathbb{1}YY\mathbb{1}\mathbb{1} + ZXX\mathbb{1}\mathbb{1} - ZYY\mathbb{1}\mathbb{1} + \mathbb{1}XX\mathbb{1}Z - \mathbb{1}YY\mathbb{1}Z + ZXX\mathbb{1}Z - ZYY\mathbb{1}Z\\
&\quad - \mathbb{1}XXZZ + \mathbb{1}YYZZ - ZXXZZ + ZYYZZ + X\mathbb{1}\mathbb{1}X\mathbb{1} - Y\mathbb{1}\mathbb{1}Y\mathbb{1} + X\mathbb{1}ZX\mathbb{1} - Y\mathbb{1}ZY\mathbb{1}\\
&\quad - X\mathbb{1}\mathbb{1}XZ + Y\mathbb{1}\mathbb{1}YZ - X\mathbb{1}ZXZ + Y\mathbb{1}ZYZ + XZ\mathbb{1}XZ - YZ\mathbb{1}YZ + XZZXZ - YZZYZ\,,\\
S_{12} &= XX\mathbb{1}\mathbb{1}X - XX\mathbb{1}ZX + XXZ\mathbb{1}X - XXZZX - YY\mathbb{1}\mathbb{1}X + YY\mathbb{1}ZX - YYZ\mathbb{1}X + YYZZX\\
&\quad - XY\mathbb{1}\mathbb{1}Y + XY\mathbb{1}ZY - XYZ\mathbb{1}Y + XYZZY - YX\mathbb{1}\mathbb{1}Y + YX\mathbb{1}ZY - YXZ\mathbb{1}Y + YXZZY\\
&\quad + \mathbb{1}\mathbb{1}XXX - \mathbb{1}ZXXX + Z\mathbb{1}XXX - ZZXXX - \mathbb{1}\mathbb{1}YYX + \mathbb{1}ZYYX - Z\mathbb{1}YYX + ZZYYX\\
&\quad + \mathbb{1}\mathbb{1}XYY - \mathbb{1}ZXYY + Z\mathbb{1}XYY - ZZXYY + \mathbb{1}\mathbb{1}YXY - \mathbb{1}ZYXY + Z\mathbb{1}YXY - ZZYXY
\end{aligned} \tag{S22}$$

(where, here and below, the superscripts denoting the different systems are omitted—we keep the order $A_I A_O B_I B_O C_I$—and the tensor products are implicit), and with the coefficients

$$\begin{aligned}
&s_1 \simeq 0.2650\,, \quad s_2 \simeq 0.6325\,, \quad s_3 \simeq -0.7641\,, \quad s_4 \simeq -0.3966\,, \quad s_5 \simeq 0.1168\,, \quad s_6 \simeq 0.2359\,,\\
&s_7 \simeq 0.0595\,, \quad s_8 \simeq 0.1764\,, \quad s_9 \simeq -0.3340\,, \quad s_{10} \simeq -0.1128\,, \quad s_{11} \simeq 0.1025\,, \quad s_{12} \simeq -0.1941\,.
\end{aligned} \tag{S23}$$

The operator $S_{ABC}^{\perp}$ is given here by (S19), with

$$
\begin{aligned}
T_1 &= \mathbb{1}Z\mathbb{1}\mathbb{1}\mathbb{1}\,, \quad T_2 = \mathbb{1}\mathbb{1}\mathbb{1}Z\mathbb{1}\,, \quad T_3 = ZZ\mathbb{1}\mathbb{1}\mathbb{1}\,, \quad T_4 = \mathbb{1}\mathbb{1}ZZ\mathbb{1}\,,\\
T_5 &= Z\mathbb{1}\mathbb{1}Z\mathbb{1}\,, \quad T_6 = \mathbb{1}Z\mathbb{1}Z\mathbb{1}\,, \quad T_7 = ZZ\mathbb{1}Z\mathbb{1}\,, \quad T_8 = Z\mathbb{1}ZZ\mathbb{1}\,, \quad T_9 = \mathbb{1}ZZZ\mathbb{1}\,, \quad T_{10} = ZZZZ\mathbb{1}\,,\\
T_{11} &= X\mathbb{1}\mathbb{1}X\mathbb{1} + X\mathbb{1}ZX\mathbb{1} - Y\mathbb{1}\mathbb{1}Y\mathbb{1} - Y\mathbb{1}ZY\mathbb{1}\,, \qquad T_{12} = XZ\mathbb{1}X\mathbb{1} + XZZX\mathbb{1} - YZ\mathbb{1}Y\mathbb{1} - YZZY\mathbb{1}\,,\\
T_{13} &= \mathbb{1}XXZ\mathbb{1} + ZXXZ\mathbb{1} - \mathbb{1}YYZ\mathbb{1} - ZYYZ\mathbb{1}\,,
\end{aligned} \tag{S24}
$$

and the coefficients

$$
\begin{aligned}
&t_1 \simeq 0.5157\,, \quad t_2 \simeq -0.2426\,, \quad t_3 \simeq 0.1482\,, \quad t_4 \simeq -0.3021\,, \quad t_5 \simeq -1.3741\,, \quad t_6 \simeq 0.4190\,, \quad t_7 \simeq 0.7865\,,\\
&t_8 \simeq -1.0662\,, \quad t_9 \simeq 0.4785\,, \quad t_{10} \simeq 0.8460\,, \quad t_{11} \simeq -0.5300\,, \quad t_{12} \simeq 0.6325\,, \quad t_{13} \simeq 0.1025\,.
\end{aligned} \tag{S25}
$$

With the witness S_{switch} thus defined, we find $\text{tr}[S_{\text{switch}} \cdot W_{\text{switch}}] = -r^*_{\text{switch}} \simeq -1.576 < 0$, as reported in the main text. Note that in order to measure the witness S_{switch}, one can decompose each of its terms in a similar way as we did in Eqs. (27)–(28) of the main text for S_{η_1,η_2} in terms of CP maps, implement them and combine the statistics in the appropriate way.

B.2 A family of witnesses for $W^{\textsf{depol}}_{\textsf{switch}}(v)$ and $W^{\textsf{deph}}_{\textsf{switch}}(v)$

Due to the geometry of the problem, with the line segments containing the processes $W^{\text{depol}}_{\text{switch}}(v)$ and $W^{\text{deph}}_{\text{switch}}(v)$ being tangent to the set of causally separable processes (see Figures 4–5 of the main text or Supplementary Figures S1–S2 below), one cannot provide a unique witness that would detect the causal nonseparability of all $W^{\text{depol}}_{\text{switch}}(v)$ or $W^{\text{deph}}_{\text{switch}}(v)$ for all $v > 0$.

Instead, we provide here a family of witnesses $S(v)$, parametrised by v. Namely, $S(v)$ and the corresponding $S^{\perp}_{ABC}(v)$ are given in the forms (S18) and (S19), with the terms S_i and T_j defined again as in Eqs. (S22) and (S24), now with the coefficients

$$
s_1 = s_2 = 1\,, \quad s_3 = s_4 = -\big(1 - \frac{v^2}{4}\big)\,, \quad s_5 = s_6 = s_8 = \frac{v^2}{4}\,, \quad s_9 = -\frac{v}{2}\,, \quad s_7 = s_{10} = s_{11} = s_{12} = 0\,, \tag{S26}
$$

and

$$
t_6 = t_7 = t_9 = t_{10} = 1\,, \quad t_1 = -t_2 = t_3 = -t_4 = -t_5/2 = -t_8/2 = 1 - \frac{v^2}{4}\,, \quad t_{11} = t_{12} = t_{13} = 0\,. \tag{S27}
$$

More explicitly, this gives (when written in the order $A_I B_I A_O B_O C_I$ for ease of notation)

$$
\begin{aligned}
S(v) = \; & |0\rangle\langle 0|^{A_I} |0\rangle\langle 0|^{B_I} \Big[\mathbb{1}^{A_O B_O C_I} + \frac{v^2}{4}(Z\mathbb{1} - \mathbb{1}Z)^{A_O B_O} Z^{C_I} - \frac{v}{2}(XX+YY)^{A_O B_O} X^{C_I} - \frac{v}{2}(XY - YX)^{A_O B_O} Y^{C_I} \Big] \\
& - \frac{1}{2}(1 - \frac{v^2}{4}) \Big[|0\rangle\langle 0|^{A_I} Z^{B_I} Z^{A_O} \mathbb{1}^{B_O} \mathbb{1}^{C_I} + Z^{A_I} |0\rangle\langle 0|^{B_I} \mathbb{1}^{A_O} Z^{B_O} \mathbb{1}^{C_I} \Big]\,.
\end{aligned} \tag{S28}
$$

One finds

$$
\text{tr}[S(v) \cdot W^{\text{depol}}_{\text{switch}}(v)] = -\Big(\frac{3-v}{2}\Big) v^2\,, \qquad \text{tr}[S(v) \cdot W^{\text{deph}}_{\text{switch}}(v)] = -v^2\,, \tag{S29}
$$

which give negative values—thus proving that $W^{\text{depol}}_{\text{switch}}(v)$ and $W^{\text{deph}}_{\text{switch}}(v)$ are causally nonseparable—for all $v > 0$.

Supplementary Figures S1 and S2 represent the witnesses $S(v)$, for various values of v, in the two-dimensional slices of the space of process matrices containing $W_{\text{switch}}, W_{\text{depol}}, \mathbb{1}^{\circ}$ and $W_{\text{switch}}, W_{\text{deph}}, \mathbb{1}^{\circ}$, respectively. Note that the witnesses $S(v)$ are not optimal to detect causal nonseparability, as they are not tangent to the set of causally separable processes. E.g., for $v = 1$, we find $\text{tr}[S(1) \cdot W_{\text{switch}}] = -1$, allowing one to prove causal nonseparability of the noisy quantum switch $W^{\mathbb{1}^{\circ}}_{\text{switch}}(v)$ (33) only down to $v > 1/2$ (to be compared to $v^*_{\text{switch}} \simeq 0.3882$ for the optimal witness). We could not find an analytical expression for optimal witnesses; nevertheless, the witnesses are good enough for our goal, which was to prove that $W^{\text{depol}}_{\text{switch}}(v)$ and $W^{\text{deph}}_{\text{switch}}(v)$ are causally nonseparable for all $v > 0$.

B.3 Restricting Alice and Bob's operations to unitaries

Here we show how to impose that Alice and Bob's operations are restricted to unitaries, and provide the witness thus obtained.

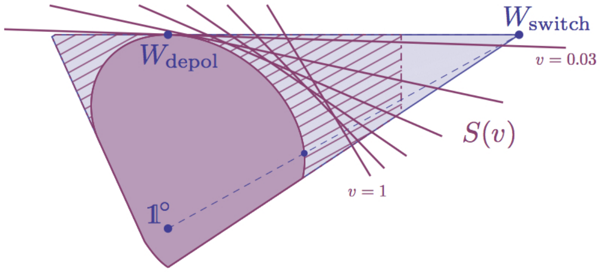

Figure S1. Two-dimensional slice of the space of process matrices containing $W_{\rm switch}, W_{\rm depol}$ and $\mathbb{1}^\circ$, as in Fig. 4 of the main text. Because the line segment containing the processes $W_{\rm switch}^{\rm depol}(v) = v\,W_{\rm switch} + (1-v)\,W_{\rm depol}$ approaches the set of causally separable processes tangentially, there is no single witness that detects their causal nonseparability for all $v>0$. Instead, one can use the family of witnesses $S(v)$ of Eq. (S28), shown here for the different values of $v = 0.03, 0.2, 0.4, 0.6, 0.8$ and 1.

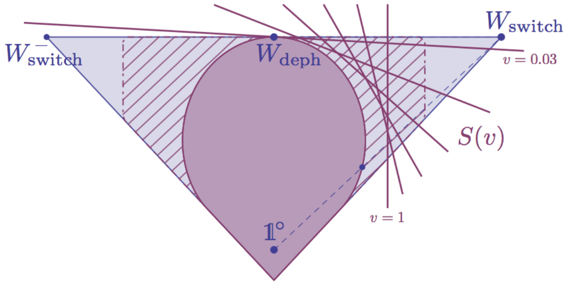

Figure S2. Two-dimensional slice of the space of process matrices containing $W_{\rm switch}, W_{\rm deph}$ and $\mathbb{1}^\circ$, as in Fig. 5. We show here the witnesses $S(v)$ for $v = 0.03, 0.2, 0.4, 0.6, 0.8$ and 1.

B.3.1 Constraints on the CJ representation of a unitary

Following the convention of,[3] the Choi-Jamiołkowski representation of a unitary operation $U : \mathcal{H}^{X_I} \to \mathcal{H}^{X_O}$ is defined as

$$M_U^{X_I X_O} = \left[(\mathbb{1} \otimes U) \, |\mathbb{1}\rangle\!\rangle\langle\!\langle\mathbb{1}| \, (\mathbb{1} \otimes U^\dagger) \right]^T , \tag{S30}$$

where $\mathbb{1}$ is the identity operator on $\mathcal{H}^{X_I}$, $|\mathbb{1}\rangle\!\rangle \equiv |\mathbb{1}\rangle\!\rangle^{X_I X_I} = \sum_j |j\rangle^{X_I} \otimes |j\rangle^{X_I} \in \mathcal{H}^{X_I} \otimes \mathcal{H}^{X_I}$ is a (nonnormalised) maximally entangled state, $\{|j\rangle^{X_I}\}$ is an orthonormal basis of $\mathcal{H}^{X_I}$, and T denotes matrix transposition in that basis.

Note first that U is a completely positive and trace-preserving map; the condition $\mathrm{tr}_{X_O} M_U^{X_I X_O} = \mathbb{1}^{X_I}$ that its CJ matrix satisfies, cf Eq. (1), can be written as

$${}_{X_O} M_U^{X_I X_O} = {}_{X_I X_O} M_U^{X_I X_O} . \tag{S31}$$

Let us furthermore calculate:

$$\mathrm{tr}_{X_I} (M_U^{X_I X_O})^T = \sum_{i,j,k} \langle i| \otimes U \cdot |j,j\rangle\langle k,k| \cdot |i\rangle \otimes U^\dagger = \sum_i U|i\rangle\langle i|U^\dagger = \mathbb{1}^{X_O} , \tag{S32}$$

from which it also follows that

$${}_{X_I} M_U^{X_I X_O} = {}_{X_I X_O} M_U^{X_I X_O} . \tag{S33}$$

B.3.2 An explicit witness made of unitaries for Alice and Bob

Solving the SDP problem (20) with CVX—in its more explicit form (55)—after replacing the constraint $S \in \mathcal{S}_V$ (or $S = L_V(S)$ in the more explicit form) by Eq. (41), we obtained numerically the witness $\tilde{S}$ and the corresponding operator $\tilde{S}_{ABC}^\perp$ of the forms (S18)–(S19), now with

$$S_1 = \mathbb{1}\mathbb{1}\mathbb{1}\mathbb{1}X, \qquad S_2 = ZZZZX, \qquad S_3 = ZZ\mathbb{1}\mathbb{1}X + \mathbb{1}\mathbb{1}ZZX,$$
$$S_4 = XXXXX + YYYYX, \qquad S_5 = XXYYX + YYXXX,$$
$$S_6 = XYXYX + YXYXX, \qquad S_7 = XYYXX + YXXYX,$$
$$S_8 = ZXZXX + ZYZYX, \qquad S_9 = ZXZYY - ZYZXY,$$
$$S_{10} = XZXZX + YZYZX, \qquad S_{11} = XZYZY - YZXZY,$$

$$
\begin{aligned}
S_{12} &= XX\mathbb{1}\mathbb{1}X - YY\mathbb{1}\mathbb{1}X + \mathbb{1}\mathbb{1}XXX - \mathbb{1}\mathbb{1}YYX\,, & S_{13} &= XY\mathbb{1}\mathbb{1}Y + YX\mathbb{1}\mathbb{1}Y - \mathbb{1}\mathbb{1}XYY - \mathbb{1}\mathbb{1}YXY\,,\\
S_{14} &= XXZZX - YYZZX + ZZXXX - ZZYYX\,, & S_{15} &= XYZZY + YXZZY - ZZXYY - ZZYXY\,,\\
S_{16} &= XZZXX - YZZYX + ZXXZX - ZYYZX\,, & S_{17} &= ZXYZY + ZYXZY - XZZYY - YZZXY\,,\\
S_{18} &= XXXYY + YXYYY - XYXXY - YYYXY\,, & S_{19} &= XXYXY + XYYYY - YXXXY - YYXYY\,,
\end{aligned}
\tag{S34}
$$

$$
\begin{aligned}
T_1 &= ZZ\mathbb{1}\mathbb{1}\mathbb{1}\,, \quad T_2 = \mathbb{1}\mathbb{1}ZZ\mathbb{1}\,, \quad T_3 = Z\mathbb{1}\mathbb{1}Z\mathbb{1}\,, \quad T_4 = \mathbb{1}Z\mathbb{1}Z\mathbb{1}\,, \quad T_5 = ZZZZ\mathbb{1}\,,\\
T_6 &= X\mathbb{1}\mathbb{1}X\mathbb{1} - Y\mathbb{1}\mathbb{1}Y\mathbb{1}\,, \quad T_7 = XX\mathbb{1}\mathbb{1}\mathbb{1} - YY\mathbb{1}\mathbb{1}\mathbb{1}\,, \quad T_8 = \mathbb{1}\mathbb{1}XX\mathbb{1} - \mathbb{1}\mathbb{1}YY\mathbb{1}\,, \quad T_9 = \mathbb{1}X\mathbb{1}X\mathbb{1} + \mathbb{1}Y\mathbb{1}Y\mathbb{1}\,,\\
T_{10} &= XXZZ\mathbb{1} - YYZZ\mathbb{1}\,, \quad T_{11} = ZZXX\mathbb{1} - ZZYY\mathbb{1}\,, \quad T_{12} = ZXZX\mathbb{1} + ZYZY\mathbb{1}\,,\\
T_{13} &= ZXXZ\mathbb{1} - ZYYZ\mathbb{1}\,, \quad T_{14} = XZZX\mathbb{1} - YZZY\mathbb{1}\,, \quad T_{15} = XZXZ\mathbb{1} + YZYZ\mathbb{1}\,,\\
T_{16} &= XXXX\mathbb{1} + YYYY\mathbb{1}\,, \quad T_{17} = XXYY\mathbb{1} + YYXX\mathbb{1}\,,\\
T_{18} &= XYXY\mathbb{1} + YXYX\mathbb{1}\,, \quad T_{19} = XYYX\mathbb{1} + YXXY\mathbb{1}\,,
\end{aligned}
\tag{S35}
$$

and with the coefficients

$$
\begin{aligned}
&s_1 \simeq -0.1396\,, \quad s_2 \simeq -0.2295\,, \quad s_3 \simeq -0.1846\,, \quad s_4 \simeq -0.1137\,, \quad s_5 \simeq -0.1262\,, \quad s_6 \simeq -0.2611\,,\\
&s_7 \simeq -0.0212\,, \quad s_8 \simeq -0.3057\,, \quad s_9 \simeq -0.2157\,, \quad s_{10} \simeq -0.1044\,, \quad s_{11} \simeq -0.0815\,, \quad s_{12} \simeq -0.1015\,,\\
&s_{13} \simeq 0.0297\,, \quad s_{14} \simeq 0.0979\,, \quad s_{15} \simeq -0.1391\,, \quad s_{16} \simeq -0.0610\,, \quad s_{17} \simeq -0.1266\,, \quad s_{18} \simeq 0.1150\,,\\
&s_{19} \simeq -0.0570\,,
\end{aligned}
\tag{S36}
$$

and

$$
\begin{aligned}
&t_1 \simeq 0.1062\,, \quad t_2 \simeq -0.1387\,, \quad t_3 \simeq -0.4969\,, \quad t_4 \simeq 0.4541\,, \quad t_5 \simeq 0.0165\,, \quad t_6 \simeq -0.5239\,,\\
&t_7 \simeq 0.0269\,, \quad t_8 \simeq -0.0134\,, \quad t_9 \simeq 0.2886\,, \quad t_{10} \simeq -0.0950\,, \quad t_{11} \simeq 0.0102\,, \quad t_{12} \simeq 0.0092\,,\\
&t_{13} \simeq 0.1254\,, \quad t_{14} \simeq 0.1128\,, \quad t_{15} \simeq -0.0439\,, \quad t_{16} \simeq 0.0994\,, \quad t_{17} \simeq 0.0251\,, \quad t_{18} \simeq -0.0680\,,\\
&t_{19} \simeq -0.1924\,.
\end{aligned}
\tag{S37}
$$

With the witness $\tilde{S}$ thus defined, we find $\mathrm{tr}[\tilde{S}\cdot W_{\mathrm{switch}}] \simeq -0.5058 < 0$. Noting that $\mathrm{tr}[\tilde{S}\cdot \mathbb{1}^\circ] = \mathrm{tr}[\tilde{S}\cdot W_{\mathrm{depol}}] = \mathrm{tr}[\tilde{S}\cdot W_{\mathrm{deph}}] = 1$, we find that $\tilde{S}$ allows one to detect the causal nonseparability of $W_{\mathrm{switch}}^{\mathbb{1}^\circ}(v)$ (33), $W_{\mathrm{switch}}^{\mathrm{depol}}(v)$ (34) and $W_{\mathrm{switch}}^{\mathrm{deph}}(v)$ (37) down to $v = 1/(1-\mathrm{tr}[\tilde{S}\cdot W_{\mathrm{switch}}]) \simeq 0.6641$, as reported in the main text.

In order to decompose the witness $\tilde{S}$ in terms of unitaries for Alice and Bob, one can apply for instance the following decomposition to each of its terms $\sigma_1^{A_I}\otimes\sigma_2^{A_O}$ and $\sigma_3^{B_I}\otimes\sigma_4^{B_O}$:

$$
\begin{aligned}
\mathbb{1}\otimes\mathbb{1} &= \tfrac{1}{2}(M_{\mathbb{1}} + M_X + M_Y + M_Z)\,, & Y\otimes X &= \tfrac{1}{2}(-M_P + M_{PX} - M_{PY} + M_{PZ})\,,\\
X\otimes X &= \tfrac{1}{2}(M_{\mathbb{1}} + M_X - M_Y - M_Z)\,, & X\otimes Z &= \tfrac{1}{2}(M_H + M_{HX} - M_{HY} - M_{HZ})\,,\\
Y\otimes Y &= \tfrac{1}{2}(-M_{\mathbb{1}} + M_X - M_Y + M_Z)\,, & Z\otimes X &= \tfrac{1}{2}(M_H - M_{HX} - M_{HY} + M_{HZ})\,,\\
Z\otimes Z &= \tfrac{1}{2}(M_{\mathbb{1}} - M_X - M_Y + M_Z)\,, & Y\otimes Z &= \tfrac{1}{2}(-M_{HP} + M_{HPX} - M_{HPY} + M_{HPZ})\,,\\
X\otimes Y &= \tfrac{1}{2}(-M_P - M_{PX} + M_{PY} + M_{PZ})\,, & Z\otimes Y &= \tfrac{1}{2}(-M_{PH} + M_{PHX} + M_{PHY} - M_{PHZ})\,,
\end{aligned}
\tag{S38}
$$

with $P = \begin{pmatrix} 1 & 0 \\ 0 & i \end{pmatrix}$ (a phase gate) and $H = \frac{1}{\sqrt{2}}\begin{pmatrix} 1 & 1 \\ 1 & -1 \end{pmatrix}$ (a Hadamard gate), and where M_U denotes the CJ matrix of the unitary U. (Note that because of Eq. (41), no term of the form $\sigma^{A_I}\otimes\mathbb{1}^{A_O}$, $\mathbb{1}^{A_I}\otimes\sigma^{A_O}$, $\sigma^{B_I}\otimes\mathbb{1}^{B_O}$ or $\mathbb{1}^{B_I}\otimes\sigma^{B_O}$ with $\sigma = X, Y$ or Z appears in the decomposition of $\tilde{S}$.) Once again, let us emphasise that such decompositions are not unique; one may choose to use a different set of unitaries to decompose $\tilde{S}$—e.g. one may want to minimise the number of different unitaries to implement (given the dimensions in play, one can do with 10 for Alice and 10 for Bob), or the total number of different terms in the decomposition of $\tilde{S}$.

References

1. Araújo, M. *et al.* Witnessing causal nonseparability. *New J. Phys.* **17**, 102001 (2015).
2. Rockafellar, R. T. *Convex Analysis* (Princeton University Press, 1970).
3. Oreshkov, O., Costa, F. & Brukner, Č. Quantum correlations with no causal order. *Nat. Commun.* **3**, 1092 (2012).
